# Greater blood volume and Hb mass in obese women quantified by the carbon monoxide-rebreathing method affects interpretation of iron biomarkers and iron requirements

**DOI:** 10.1038/s41366-018-0127-9

**Published:** 2018-06-15

**Authors:** Ana C. Cepeda-Lopez, Michael B. Zimmermann, Sophia Wussler, Alida Melse-Boonstra, Nicole Naef, Sandro Manuel Mueller, Marco Toigo, Isabelle Herter-Aeberli

**Affiliations:** 1 Division of Human Nutrition, Wageningen University (WU), Wageningen, The Netherlands; 2 Health Sciences Division, University of Monterrey (UDEM), Monterrey, Mexico; 3 Laboratory of Human Nutrition, ETH Zürich, Zürich, Switzerland; 4 University Hospital Balgrist, Balgrist Move>Med, Zurich, Switzerland; 5 Department of Neurology, University Hospital Zurich, Zurich, Switzerland; 6 Institute of Human Movement Sciences, ETH Zurich, Zurich, Switzerland; 7 University Hospital Balgrist, Laboratory for Muscle Plasticity, Zurich, Switzerland

**Keywords:** Risk factors, Physiology

## Abstract

**Background/objective:**

Iron deficiency (ID) is common in overweight and obese individuals (OW/OB) but the mechanism is uncertain. Greater blood volume (BV) in OW/OB may increase hemoglobin (Hb) mass and iron requirements, and confound iron biomarkers by hemodilution. Quantification of BV/PV changes in OW/OB is challenging and a formula to estimate BV/PV based on anthropometric indices would be valuable. In normal weight (NW) and OW/OB women, we aimed at: (1) measure BV and assess whether differences in BV affect concentrations and total circulating mass of Hb and iron biomarkers; (2) develop an algorithm describing BV in OW/OB.

**Subjects/methods:**

In a cross-sectional study, we measured BV in NW, OW, and OB non-anemic women (*n* = 62) by using the carbon monoxide-rebreathing method, body composition by dual energy X-ray absorptiometry, and iron and inflammatory status.

**Results:**

OW and OB women had 11 and 16% higher mean BV and PV compared to NW (*P* < 0.05), respectively. In OW/OB compared to NW, total circulating masses of IL-6, hepcidin, Hb, and sTfR were higher, while total mass of serum iron was lower (for all, *P* < 0.05). An equation including height, body mass and lean mass to estimate BV in all BMI groups (*R*^2^ = 0.76).

**Conclusion:**

An equation based on anthropometric indices provides a good estimate of increased BV in OW/OB women. In OW/OB women, there is an increase in Hb mass that likely increases iron requirements for erythropoiesis and circulating TfR mass. At the same time, higher hepcidin concentrations may lower serum iron mass. Both these mechanisms may increase risk for ID in OW/OB women.

## Introduction

Body mass index (BMI) is negatively correlated with serum iron and positively correlated with serum transferrin receptor (sTfR) in children and adults [1]. Compared to normal weight individuals (NW), iron deficiency (ID), defined by these biomarkers, is more common in overweight and obese individuals (OW/OB) [2–12]. The etiology of ID in OW/OB is likely multifactorial [1]. Adiposity-related inflammation increases serum hepcidin which reduces iron absorption from duodenal enterocytes increasing risk for absolute ID [13, 14]. At the same time, high serum hepcidin may reduce recycling of iron from senescent erythrocytes by macrophages, reducing the amount of circulating serum iron and thereby limiting erythropoiesis [15], causing a ‘relative’ ID despite normal body iron stores [3]. Total blood volume (BV) increases in OW/OB to meet the increased metabolic demand of the extra body weight, due to greater perfusion of adipose tissue and the attendant increase in lean body mass [16]. Thus, OW/OB may have a greater iron requirement due to their larger RBC and hemoglobin mass, increasing risk of ID. Finally, increased plasma volume (PV) in OW/OB could reduce serum iron through hemodilution and contribute to hypoferremia [4, 7–9, 11, 12, 15, 17]. The effects of increased BV and PV on biomarkers of iron status in OW/OB is uncertain.

Several simple equations exist for the estimation of BV in individuals but they are mainly based on data from NW subjects [18–20]. Although they have been applied in OW/OB and yielded estimates of PV increases of ≈10–22% [21, 22], their validity in OW/OB is uncertain, because adipose tissue is relatively poorly perfused compared to lean tissue. Traditional methods for the exact determination of BV are challenging because they are invasive and may use radioisotopes [19, 20]. The carbon monoxide (CO)-rebreathing method is a valid and reliable method for assessing BV that is minimally invasive and involves no radioactivity [23]. This method would be ideal to estimate the increase in BV in OB; the resulting BV measures could be used to estimate total circulating mass of the iron status biomarkers to clarify the etiology of ID in OB. In addition, the CO-rebreathing method may allow the development of a validated new equation for the determination of BV in overweight and obese individuals from simpler anthropometric measures.

Therefore, our study aims were: (1) to quantitatively measure BV and PV, and iron and inflammatory status biomarkers in OW/OB women and NW women; (2) use the measures in [1] to calculate circulating masses of hemoglobin and iron biomarkers and compare them in OW/OB versus NW women; and (3) develop a new equation based on anthropometric indices to allow reliable estimation of BV in OW/OB women.

## Materials/subjects and methods

### Subjects

This study was part of a larger study that investigated the influence of body mass and fat on iron absorption in NW, OW, and OB women where the sampling methodology and response rates are published in detail [24]. Briefly, the sample-size calculation was based on the expected differences in iron absorption of 35%, a power of 80%, and an a-level of 0.05 resulted in a required sample size of 22 subjects/group. With an expected 10% noncompletion rate taken into account, we aimed for a sample size of 25 subjects/group.

The subjects in this study were 64 women: 24 with NW (NW: BMI 18.5–24.9 kg/m^2^); 20 with overweight (OW, BMI 25–29.9 kg/m^2^); and 20 with obesity (OB: BMI 30–39.9 kg/m^2^). At screening, we informed women about the study aims, procedures, and associated risks. Inclusion criteria for the study were: [1] female; [2] age 18–45 y; [3] pre-menopausal; [4] BMI 18.5–39.9 kg/m^2^; [5] no chronic illness that could influence iron or inflammatory status other than obesity; [6] non-smoking or smoking <2 cigarettes/week (in the latter group, not smoking three days prior to the first measurement and during the study); [7] nonpregnant and not planning a pregnancy. We collected subject information using a questionnaire, measured body mass and height to determine BMI, and performed a pregnancy test. The ethics committees of ETH Zurich and the Canton of Zurich approved the study, and we registered it at clinicaltrials.gov (NCT01884506). We obtained written informed consent from all participants.

At screening, we obtained a fasting blood sample via forearm venipuncture for analysis of hemoglobin, and iron and inflammation status. We determined body composition and BV by using dual energy X-ray absorptiometry (DXA) and CO-rebreathing, respectively. We scanned all participants on a DXA GE Healthcare Lunar according to the manufacturer’s specifications (DXA; Lunar iDXA, GE Healthcare, Madison, WI, USA). We performed the scan analysis using the GE encore software version 11.40.004. To minimize fluctuations in BV, we performed all measurements, if possible, between 7 and 14 days after the beginning of the last menstrual cycle. The optimized CO rebreathing method [25] is a routinely-applied, minimally invasive, reliable method for assessing BV [26]. This method has been described in detail elsewhere [23, 25, 27]. Briefly, 15 min after the subjects adopted a stationary seated position, we obtained baseline venous samples via forearm venipuncture (2 ml collected in an EDTA tube) for measurement of hemoglobin (Hb) and hematocrit (Hct). All blood samples were immediately analyzed using a spectrophotometer for the blood gas determination (ABL 700 Serie, Radiometer A/S, Copenhagen, Denmark). Furthermore, we obtained capillary blood samples from the earlobe (35 μL in pre-heparinized glass capillary tubes) in triplicate at baseline and at 6, 7, and 8 min after starting the rebreathing procedure. We measured percent carboxyhemoglobin saturation (HbCO%) by using a blood gas analyzer. The mean value of the measurements at 6 and 8 min was taken as the plateau value after CO-rebreathing with the sample at minute 7 as a backup. Total Hb mass was calculated as described previously [28], using a slightly different correction for loss of CO to myoglobin (0.3%/min of administered CO) [25]. We administered a bolus of chemically pure CO of 0.8 ml/kg body mass during the first inspiration from a closed spirometric system (Blood tec GbR, Bayreuth, Germany) and this was rebreathed for 110 s together with a small amount of oxygen (4 l). To verify that no gas was leaking during the CO rebreathing procedure, the entire apparatus as well as the mouth piece and nose-clip were checked using a portable CO gas analyzer (Dräger PAC 7000; Dräger Safety; Lübeck, Germany) with a parts-per-million sensitivity to monitor local CO levels. The analyzer was also used to calculate end-tidal CO concentration before the CO-rebreathing and after the onset of the rebreathing procedure with the subject wearing a nose-clip and then blowing into a mouthpiece until the maximal value of CO was observed and recorded. We also measured the amount of CO remaining in the spirometer after rebreathing with the portable CO gas analyzer. We used the measured parameters to first calculate total Hb mass and from this derived blood, plasma, and red blood cell volume using equations published previously [23, 25, 28]. We verified the reproducibility of the method by a test–retest correlation analysis and quantified the typical error of measurement by calculation. For this assessment, we applied the same optimized CO rebreathing method to the same subjects (*n* = 8) on two separate occasions. The typical error was 1.2% with an *R*^2^ = 0.98 [29].

### Laboratory analysis

We measured Hb concentration by using a Coulter counter (Beckman Coulter, Krefeld, Germany) with 3-level quality-control material (Liquichek; Bio-Rad, Irvine, CA) on the day of blood collection. We measured serum iron and total-iron binding capacity (TIBC) by using a colorimetric method as described previously [30]. We measured sTfR, serum ferritin (SF), high-sensitivity C-reactive protein (CRP) and alpha 1 glycoprotein (AGP) by using a multiplex immunoassay [31], and interleukin 6 (IL-6) by using a Quantikine ELISA kit (R&D systems, Minneapolis, MN). We used correction factors to remove the effects of inflammation on SF [32] and then calculated body iron stores using the formula of Cook et al. [33]. We calculated the total mass of circulating biomarkers for each women by multiplying the plasma concentration times the measured PV [21, 22].

### Statistical analysis

We performed statistical analyses using IBM SPSS Version 20 (IBM Company, Armonk, NY, USA). We checked data for normality by visual observation and by using the Kolmogorov–Smirnov and Levene’s normality tests. Non-normally distributed data were logarithmically transformed for statistical analysis. The mean (±s.d.) for normally distributed data, geometric mean (95% confidence interval) for data with normal distribution after log-transformation or median (interquartile range) for non-normally distributed data even after log-transformation values for each parameter were determined. We applied nonparametric tests for data that remained non-normally distributed after logarithmic transformation. We assessed differences between NW, OW, and OB using analysis of variance (ANOVA) with post hoc Bonferroni correction and Kruskal–Wallis test, followed by Mann–Whitney *U* test, as appropriate. To study associations between continuous variables, we used bivariate Pearson’s or Spearman correlations and multiple linear regression models with adjustments for confounding variables. Differences were considered significant at *P*-values < 0.05.

To create a new equation for the calculation of BV, we included height, body mass, body surface area (BSA), BMI, total lean, and fat mass in a regression analysis, and screened for those that best estimated measured BV. *R*^2^ was used to determine the accuracy of this approximation.

## Results

**Fig. 1:**
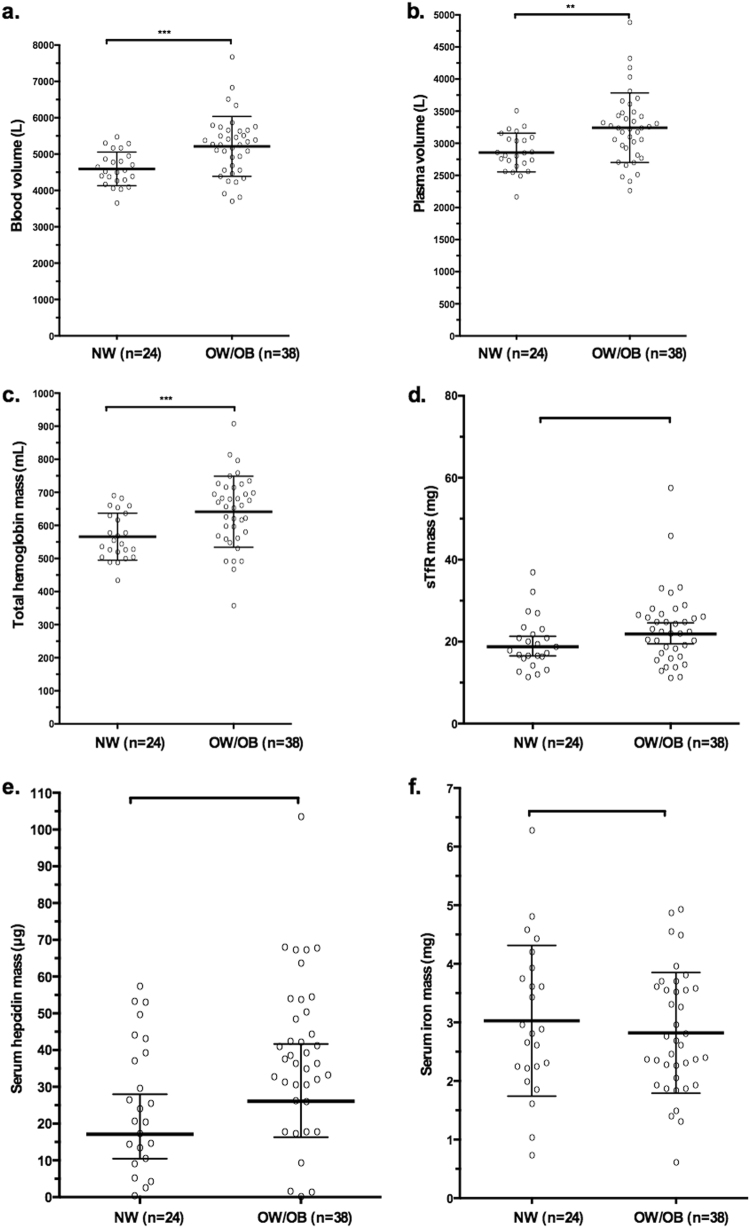
Absolute blood (**a**), and plasma volume (**b**), as well as masses of total Hb (**c**), sTfR (**d**), serum hepcidin (**e**), and serum iron (**f**) in NW and OW/OB. Error bars are means ± SDs (for absolute blood, and plasma volume, total mass of Hb, serum iron and hepcidin) or geometric means (95% CIs) (for sTfR). Comparisons between NW and OW/OB was assessed with independent samples *t*-test. NW normal-weight group (*n* = 24), OB/OW overweight/obese group (*n* = 38). *P < 0.05; **P < 0.01; ***P < 0.001

Of the 64 women enrolled, two subjects (1 OW and 1 OB) dropped out of the study; 62 women completed the study and were included in the analyses. Age, anthropometry, body composition, and inflammatory biomarkers are shown in Table 1, while BV and PV, Hb mass and iron biomarkers are shown in Table 2. OW and OB were significantly older, heavier, and had greater adipose mass than NW (*P* *<* 0.05); OB had significantly greater lean body mass than NW (*P* *<* 0.05). Total BV and RBC volume (mL) were greater in OB and OW compared to NW (for all, *P* *<* 0.05) while blood and RBC volume in mL/kg were significantly lower in OB and OW compared to NW (for all, *P* *<* 0.05*)*. Mean PV was higher by 11% in the OW (*P* *=* 0.07) and 16% in the OB (*P* *=* 0.010) compared to the NW women. PV in mL/kg was lower in OB and OW compared to NW (*P* < 0.001*)*. Although Hb concentration (g/L) was not significantly different among the 3 groups, total Hb mass (g) was 11% greater in OW (*P* *=* 0.015) and 16% greater in OB (*P* *=* 0.005) compared to NW, while Hb mass in g/kg was 14% lower in OW and 22% lower in OB (for both, *P* *<* 0.001) compared to NW. Serum concentrations and circulating masses of CRP, IL-6, AGP and hepcidin were greater in OB versus NW (*P* *<* 0.05). There were no significant differences in concentrations of sTfR or SF among the four groups. However, total masses of sTfR and SF were greater in OW/OB compared to NW (*P* *=* 0.07 and *P* *=* 0.03, respectively). In OW/OB compared to NW, concentration of serum iron was lower (*P* *=* 0.025) and total serum iron mass was lower (*P* *=* 0.014). In OW/OB compared to NW, concentrations of serum TIBC was lower (*P* *=* 0.04) but total mass of TIBC was not. Transferrin saturation was lower in OW/OB, but this was not statistically significant (Fig. 1).

**Table 1:** Anthropometric characteristics and inflammatory biomarkers in normal weight (NW), overweight (OW), and obese (OB) Swiss women (*n* = 62)

	NW	OW	OB	OW/OB
*n*	24	19	19	38
Age (y)^a^	23 (22, 26)^d,e^	26 (22, 29)	27 (23, 33)	27 (23, 31)
Body mass (kg)^b^	61.8 ± 7.2^d,e,f^	78.7 ± 7.3^d^	89.0 ± 12.0	83.9 ± 11.1
BMI (kg/m^2^)^b^	21.9 ± 1.9^d,e,f^	27.3 ± 1.5^d^	32.8 ± 2.8	30.1 ± 3.6
Total body fat %^b^	29.8 ± 4.8^d,e,f^	40.1 ± 4.4^d^	45.8 ± 4.2	43.0 ± 5.1
Fat mass (kg)^b^	18.0 ± 4.6^d,e,f^	30.9 ± 5.1^d^	40.1 ± 8.1	35.5 ± 8.1
Lean mass (kg)^b^	42.9 ± 4.9^d,e^	46.2 ± 4.8	47.1 ± 5.0	46.7 ± 4.8
CRP (mg/dL)^b^	1.05 (0.60, 1.85)^d,e^	2.23 (1.23, 4.02)	3.22 (1.77, 5.86)	2.68 (1.78, 4.01)
Total CRP (mg)^c^	29.87 (15.4, 53.5)^d,e^	70.1 (34.1,143.5)	104.6 (46.9, 232.6)	85.6 (48.1,151.6)
IL-6 (pg/mL)^c^	0.69 (0.53, 0.91)^d,e^	0.75 (0.57, 1.0)^d^	1.25 (1.03, 1.53)	0.97 (0.81, 1.17)
Total IL-6 (ng)^c^	1.95 (1.36, 2.63)^d,e^	2.38 (1.58, 3.57)^d^	4.05 (2.73, 6.07)	3.1 (2.19, 4.42)
AGP (g/L)^b^	0.79 ± 0.2^d,e^	0.90 ± 0.3	1.00 ± 0.3	0.95 ± 0.3
Total AGP (g)^b^	2.24 ± 0.53^d,e,f^	2.86 ± 0.97	3.14 ± 1.17	3.10 ± 0.82

Differences between NW, OW, and OB were assessed using one-way ANOVA with post hoc Bonferroni correction and Kruskal–Wallis test followed by independent samples Mann–Whitney *U* test corrected for multiple comparisons. Difference between NW and OW/OB was assessed with independent samples *t*-test. *P*-values 0.05 were considered significant

*NW* normal weight (BMI 18.5–24.9 kg/m^2^), *OW* overweight (BMI 25–29.9 kg/m^2^), *OB* obese (BMI 30–39.9 kg/m^2^), *OW/OB* overweight and obese (BMI 25–39.9 kg/m^2^), *BMI* body mass index, *CRP* C-reactive protein, *IL6* interleukin-6, *AGP* alpha 1 glycoprotein, *IQR* interquartile range

Values are: ^a^median (IQR), ^b^mean (±s.d.) and ^c^geometric mean (95% confidence interval)

^d^Significantly different from obese

^e^Significantly different from OW/OB

^f^Significantly different from overweight

**Table 2:** Hematologic characteristics and iron status biomarkers in normal weight (NW), overweight (OW), and obese (OB) Swiss women (*n* = 62)

	NW	OW	OB	OW/OB
*n*	24	19	19	38
Blood volume (mL/kg)^b^	74.9 ± 5.8^d,e,f^	64.3 ± 5.3^e^	59.4 ± 7.1	61.8 ± 6.7
Blood volume (L)^b^	4.59 ± 0.46^d,e,f^	5.10 ± 0.58	5.33 ± 1.01	5.21 ± 0.82
Plasma volume (mL/kg)^b^	46.6 ± 4.8^d,e,f^	40.1 ± 4.1	36.9 ± 4.9	38.5 ± 4.7
Plasma volume (L)^b^	2.86 ± 0.30^d,e,f^	3.17 ± 0.40	3.31 ± 0.66	3.24 ± 0.54
RBC (mL/kg)^b^	28.2 ± 2.1^d,e,f^	24.3 ± 2.6	22.4 ± 2.8	23.4 ± 2.8
RBC volume (L)^b^	1.74 ± 0.22^d,e,f^	1.93 ± 0.26	2.02 ± 0.39	1.97 ± 0.33
Hb (g/dL)^b^	13.5 ± 0.9	13.7 ± 1.2	13.7 ± 1.0	13.7 ± 1.1
Hb mass (g/kg)^b^	9.2 ± 0.7^d,e,f^	7.9 ± 0.8	7.3 ± 0.9	7.6 ± 0.9
Total Hb (g)^b^	566 ± 71^d,e,f^	627 ± 85	656 ± 126	641 ± 107
Serum iron (μg/mL)^b^	1.06 ± 0.44^e^	1.01 ± 0.35	0.75 ± 0.27	0.89 ± 0.33
Total serum iron (mg)^b^	3.02 ± 1.28^e^	3.14 ± 0.98^e^	2.50 ± 1.00	2.82 ± 1.03
TIBC (μg/mL)^b^	3.68 ± 0.66^e^	3.62 ± 0.66	3.17 ± 0.66	3.39 ± 0.69
Total TIBC (mg)^b^	10.5 ± 2.28	11.6 ± 3.00	10.3 ± 2.18	10.9 ± 2.66
Transferrin saturation (%)	29.8 ± 13.4	29.1 ± 12.1	24.6 ± 8.66	26.8 ± 10.6
sTfR (mg/L)^c^	6.61 (5.86, 7.45)	6.69 (5.82, 7.68)	7.00 (5.90, 8.30)	6.84 (6.16, 7.59)
Total sTfR (mg)^c^	18.8 (15.0, 21.5)^f^	21.1 (16.1, 27.4)	22.8 (15.6, 33.0)	21.9 (16.6, 28.7)
SF (µg/L)^c^	50.6 (40.2, 63.4)	59.9 (41.3, 86.8)	62.8 (45.4, 86.9)	61.35 (48.5, 77.6)
Total SF (mg)^c^	0.14 (0.10, 0.18)^f^	0.19 (0.11, 0.31)	0.20 (0.12, 0.35)	0.20 (0.13, 0.29)
Body iron (mg/kg)^b^	5.64 ± 2.70	5.92 ± 3.31	5.46 ± 3.10	5.70 ± 3.17
Serum hepcidin (ng/mL)^b^	9.20 ± 6.44	11.36 ± 6.73	12.48 ± 5.90	11.92 ± 6.3
Total hepcidin (µg)^b^	25.5 ± 17.6	35.4 ± 21.1	41.2 ± 21.5	38.3 ± 21.2

Differences between NW, OW, and OB were assessed using one-way ANOVA with post hoc Bonferroni correction and Kruskal–Wallis test followed by independent samples Mann–Whitney *U* test corrected for multiple comparisons. Difference between NW and OW/OB was assessed with independent samples *t*-test. *P*-values < 0.05 were considered significant

*NW* normal weight (BMI 18.5–24.9 kg/m^2^), *OW* overweight (BMI 25–29.9 kg/m^2^), *OB* obese (BMI 30–39.9 kg/m^2^), *OW/OB* overweight and obese (BMI 25–39.9 kg/m^2^), *Hb* Hemoglobin, *TIBC* total iron binding capacity, *sTfR* soluble transferrin receptor, *IQR* interquartile range.

Values are: ^a^median (IQR), ^b^mean (±s.d.), and ^c^geometric mean (95% confidence interval)

^d^Significantly different from overweight

^e^Significantly different from obese

^f^Significantly different from OW/OB

**Fig. 2:**
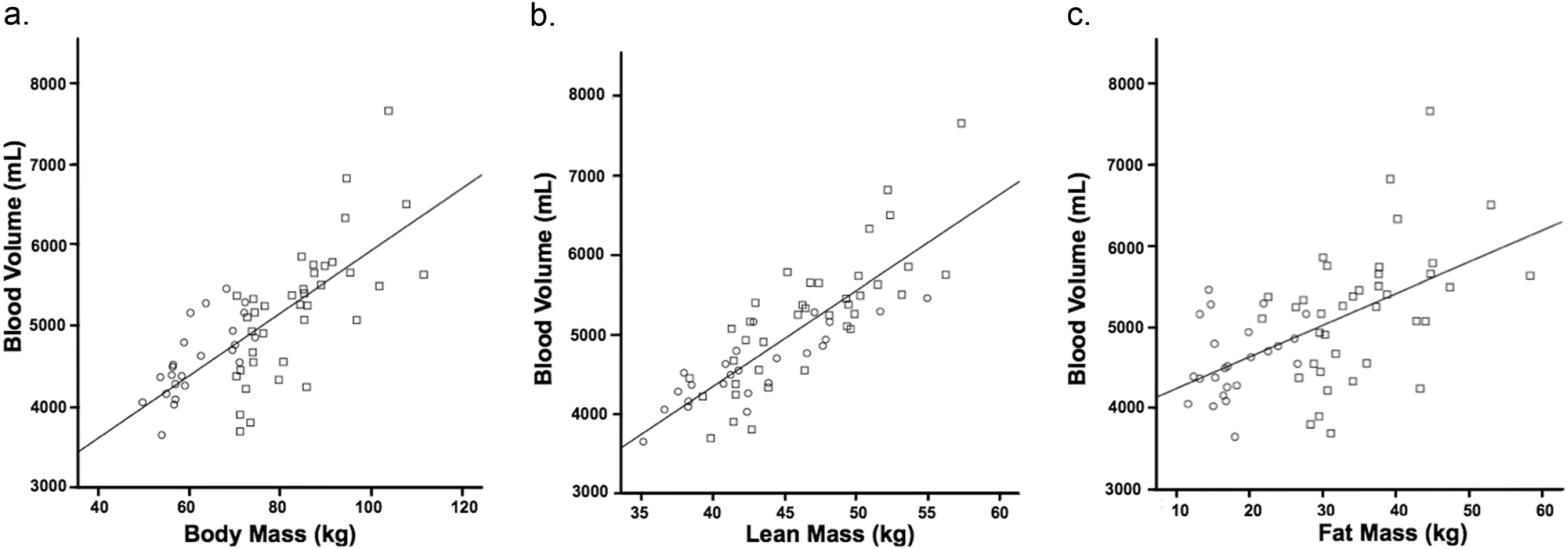
Correlations between absolute blood volume and (**a**) body mass; (**b**) lean mass and; (**c**) fat mass

Correlations between body mass, lean and fat mass, and absolute BV are presented in Fig. 2. BV was more closely correlated with body mass (*R* = 0.74, *P* *<* 0.001) and total lean mass (*R* = 0.83, *P* *<* 0.001), than with fat mass (*R* = 0.55, *P* *<* 0.001), waist circumference (*R* = 0.52, *P* *<* 0.001), height (*R* = 0.58, *P* *<* 0.001) or BMI (*R* = 0.50, *P* *<* 0.001).

Correlations between relative and absolute iron status indicators with anthropometry, body composition, BV and inflammation biomarkers are shown in Table 3. Serum iron was positively associated with hemoglobin (*R* = 0.32, *P* *=* 0.01), body iron stores (*R* = 0.38, *P* *=* 0.002), SF (*R* = 0.34, *P* *=* 0.01), and negatively associated with BMI (*R* = −0.37, *P* *=* 0.003), body mass (*R* = −0.36, *P* *=* 0.004), fat mass (*R* = −0.38, *P* *=* 0.002), AGP (*R* = −0.27, *P* *=* 0.04), BV (*R* = −0.20, *P* *=* 0.11), and PV (*R* = −0.25, *P* *=* 0.05). Mass of serum iron was positively associated with circulating mass of SF (*R* = 0.34, *P* *=* 0.01) and hepcidin mass (*R* = 0.27, *P* *=* 0.03). Moreover, mass of SF was positively associated with hemoglobin mass (*R* = 0.45, *P* *<* 0.001), mass of inflammatory markers (mass of CRP: *R* = 0.47, *P* *<* 0.001; and mass of AGP: *R* = 0.44, *P* *<* 0.001), BMI (*R* = 0.35, *P* *=* 0.005), body mass (*R* = 0.40, *P* *=* 0.001), and fat mass (*R* = 0.40, *P* = 0.001) but not with total lean mass (*R* = 0.20, *P* *=* 0.11). In multiple linear regression analysis, total body fat (*R*^2^ = 0.31, *β* = −0.47, *P* *<* 0.001) but not lean mass (*R*^2^ = 0.31, *β* = 0.03, *P* *=* 0.84) was a significant negative predictor of serum iron when corrected for body iron stores. Moreover, BV was a significant negative predictor for serum iron (*R*^2^ = 0.18, *β* = −0.29, *P* < 0.05) when corrected for body iron stores. Baseline BMI was a significant predictor for total serum iron mass (*R*^2^ = 0.21, *β* = −0.27, *P* = 0.03) and TfR mass (*R*^2^ = 0.56, *β* = 0.31, *P* = 0.001) but not for total serum TIBC mass (*R*^2^ = 0.12, *β* = 0.06, *P* = 0.64) when corrected for body iron stores. PV was a significant predictor for total TIBC mass (*R*^2^ = 0.40, *β* = 0.53, *P* < 0.001) and TfR mass (*R*^2^ = 0.64, *β* = 0.42, *P* < 0.001) but not for total serum iron mass (*R*^2^ = 0.15, *β* = 0.08, *P* = 0.50), when corrected for body iron stores.

**Table 3:** Univariate correlations between relative and absolute iron status biomarkers and anthropometry, body composition, and inflammation biomarkers in a group of women (*n* = 62) with BMI between 18.5 and 39.9 kg/m^2^

	Anthropometry and body composition				Inflammation biomarkers					
Variable	BMI	Lean mass	Fat mass	Blood Volume	CRP	IL6	AGP	Mass CRP	Mass IL6	Mass AGP
Hb	0.12	0.06	0.14	0.12	0.00	−0.09	0.28*	−0.03	−0.14	0.14
Hb Mass	0.50***	0.81***	0.56**	0.78**	0.20	0.00	0.16	0.28*	0.18	0.52***
Serum iron	−0.37**	−0.16	−0.38**	−0.34**	−0.09	−0.16	−0.27*	−0.11	−0.21	−0.32*
TIBC	−0.34**	−0.16	−0.37**	−0.34**	0.01	−0.08	−0.35**	−0.02	−0.11	−0.38**
Serum ferritin	0.25*	0.02	0.30*	0.25*	0.36**	0.06	0.19	0.38**	0.11	0.28*
TfR	0.02	−0.12	−0.06	−0.11	−0.12	0.16	0.03	−0.10	0.16	0.02
Hepcidin	0.19	−0.06	0.22	0.14	0.18	0.02	0.29*	0.15	−0.01	0.21
Mass serum iron	−0.20	0.12	−0.20	−0.07	0.12	−0.013	−0.25	0.34	−0.10	−0.12
Mass TIBC	0.00	0.36**	0.00	0.17	0.18	0.00	−0.27*	0.24	0.12	0.02
Mass sTfR	0.19	0.17	0.15	0.18	0.00	0.20	0.08	0.06	0.29*	0.25*
Mass SF	0.35**	0.20	0.40**	0.41**	0.42**	0.08	0.23	0.47**	0.19	0.44**
Mass Hepcidin	0.31*	0.15	0.35**	0.34**	0.25*	0.03	0.31*	0.26*	0.08	0.38**

Spearman’s rho coefficient or Pearson’s coefficient

*BMI* body mass index, *Hb* Hemoglobin, *TIBC* total iron binding capacity, *TfR* soluble transferrin receptor, *SF* serum ferritin, *CRP* C-reactive protein, *IL6* interleukin-6, *AGP* alpha 1 glycoprotein

*P < 0.05; **P < 0.01; ***P < 0.001

Two equations for the estimation of measured BV were created using stepwise regression with a forward selection of covariates. They are shown below and their performance is shown in Table 4.

**Table 4:** Blood volume calculated with CO-rebreathing method and derived from linear regression equations with body mass and height (Formula 1) or body mass, height and lean mass (Formula 2) as predictor variables in women (*n* = 62) with body mass index (BMI) 18.5 to 39.9 kg/m^2^

	Normal-weight (*n* = 24)			Overweight/obese (*n* = 38)			Total population (*n* = 62)		
	Mean BV	SD	*R* ^2^	Mean BV	SD	*R* ^2^	Mean BV	SD	*R* ^2^
	mL	mL		mL	mL		mL	mL	
Calculated CO-rebreathing	4593	463	–	5212	823	–	4973	764	–
Formula 1	4557	448	0.52	5235	593	0.69	4973	633	0.69
Formula 2	4575	531	0.75	5221	627	0.75	4971	668	0.76

Formula 1: (4698.8 × height) + (32.5 × body mass)−5342.1; Formula 2: (2686.7 × height) + (18.9 × body mass) + (66.0 × lean mass)−3932.3. Height in centimeters and body mass in kilograms. The accuracy of the regression equation was assessed using *R*^2^, the coefficient of determination. *R*^2^ presents the proportion of the observed variance that can be explained through the equation

*BV* blood volume, *CO* carbon monoxide

(1) Blood volume (mL) = 4698.8 × height (m) + 32.5 × body mass (kg)−5342.1 (*R*^2^ = 0.69, *P* < 0.01)

(2) Blood volume (mL) = 2686.7 × height (m) + 18.9 × body mass (kg) + 66 × lean mass (kg)−3932.3 (*R*^2^ = 0.76, *P* < 0.01)

## Discussion

In this study, the calculation of individual BV and PV allowed us to quantify and compare total circulating mass of Hb, iron and inflammatory biomarkers among NW and OW/OB. Although Hb concentration was not significantly different among the 3 groups, total Hb mass was 11–16% greater in OW/OB compared to NW. This significant increase in total Hb mass in OW/OB likely increases the iron requirement in this group compared to NW, and may increase risk for deficiency if iron intakes, or iron absorption, are low. The 23% greater total sTfR mass in OW/OB may be due to a higher level of erythropoiesis to support the higher total Hb mass and/or iron deficient erythropoiesis in OW/OB [1]. In turn, iron deficient erythropoiesis may be caused by a lack of circulating iron available for marrow uptake for erythropoiesis, as reflected in the 17% lower total serum iron mass and transferrin saturation in OW/OB compared to NW. Finally, lower serum iron in OW/OB is likely due to higher concentrations and total mass of serum hepcidin (increased by 62% compared to NW) probably triggered by higher concentrations and total mass of IL-6 (increased by 108% compared to NW), which could be originating from the higher fat mass in OW/OB [34].

Similar to our findings, previous studies observed lower serum iron concentrations but no differences in Hb concentrations in obese women compared to peers with NW [7, 8, 12, 17] and have suggested that the hypoferremia of obesity might be due to hemodilution [1]. Our data indicate that the total mass of serum iron is lower in OW/OB compared to NW, and transferrin saturation is also lower, suggesting that the hypoferremia is not explained only by hemodilution. However, higher BV predicted lower serum iron and thus it is likely that hemodilution at least partly contributes to the observed hypoferremia. Our results confirm previous studies showing that OW/OB women have increased levels of inflammation [3, 10, 12, 35] as reflected in sharply higher serum concentrations and total mass of IL-6; IL-6 stimulates hepatic synthesis [34], and in our study, serum and total mass of hepcidin was much higher in OW/OB compared to NW and was positively correlated with IL-6. High concentrations of serum hepcidin in OW/OB stimulated by adiposity-related inflammation likely impair iron absorption and, combined with the increased iron requirement due to the increase in Hb mass, increase risk for ID in OW/OB. Weight loss in obese subjects can reduce inflammation, lower serum hepcidin, and improve iron absorption [35].

In our study, OW/OB women had higher absolute BV values. In OW/OB individuals, adipose tissue comprises a substantial proportion of total body mass. The enlarged vascular beds of adipose tissue are less vascularized than other tissues [36]. Consequently, perfusion of the increased amount of adipose tissue alone does not explain the increase in BV since adipose tissue is oligemic compared with lean tissue. Moreover the modulation of blood flow in adipose tissue typically prevents the redistribution of the extra volume present in the interstitial space of adipose tissue into circulation [37]. This implies that the turnover rates of blood in obesity are low. However, the expansion of fluid volumes at the extracellular compartment results in hypervolemia. As compared to NW women, OW/OB women have a lower total body water per unit of weight and a higher extracellular water per unit of total body water [38]. Hyperinsulinaemia, characteristic of OW/OB, can contribute by causing sodium retention in the diluting segment of the distal nephron with consequent water retention [39]. The water retention in OW/OB increases absolute volume that is predominantly redistributed in the cardiopulmonary area, leading to augmented venous return and cardiac output [40].

Typically, BV has been expressed in terms of mL per kg of total body mass (mL/kg), using fixed ratios for normal values, with different ratios for men and women. Estimated BV calculations are usually performed with the formula: body mass (kg)×average blood volume (mL/kg). In the clinical practice BV is estimated to be 65–75 mL/kg (adult men, 75 mL/kg; and women, 65 mL/kg). However similar to our results different studies have shown that total BV and its components, plasma and red blood cells, decreases per unit body mass with increasing degree of obesity [41–43]. In a study comparing BV in OB and NW subjects, there was a lower mean unit BV of 46 mL/kg in OB compared to 86 mL/kg in NW [44]. In our study, BV was more closely correlated with body mass and total lean mass than with fat mass, waist circumference or BMI.

Estimation of BV in OW/OB is challenging since BV is not a constant fraction of body mass. Currently existing formulas for the calculation of BV [18, 20, 45–49] include different parameters that could influence BV such as body size (height, body mass and BSA), body composition (fat or lean body mass) or combinations of these parameters as predictors. An advantage of the equations for calculating BV that we propose in this study is that they were derived from a population of women with a wide BMI range. Moreover, inclusion of lean mass in the formula contributes to a better estimation of BV in OW/OB. Our interest in calculating BV in OW/OB was to be able to correctly interpret absorption data from studies conducted in OW/OB, for which BV calculation is necessary. The assessment of BV is however also an important tool in clinical medicine for the evaluation of several disorders or diseases. Additionally, changes in BV affect the distribution of numerous drugs. Increases in BV can affect peak plasma concentration, clearance and elimination half-life of many anesthetic agents [50]. Thus, doses of drugs are scaled based on the individual patient characteristics including body composition and BV [51].

The strengths of the present study include: (1) the assessment of BV using the CO-rebreathing method in women with a wide range of BMI, including an assessment of the reproducibility of the CO-rebreathing method; (2) co-measurement of lean and fat mass using DXA; and (3) measurement of a number of different biomarkers of iron and inflammation status including serum iron, sTfR, TIBC, transferrin saturation, hepcidin, and IL-6 and individual calculation of their circulating masses. A limitation of the study is that we have only studied Swiss women; whether the findings and the derived equations apply to men and to other ethnic groups is uncertain. Also, the steps involved in BV measurement using the CO-rebreathing method present potential opportunities for error. However, parameters that could influence the BV measurements were standardized to minimize measurement errors, and the reproducibility of the method was verified by a test–retest correlation analysis and the CV was low (1.2%). Due to the small sample size and a cross-sectional design, our results should be interpreted carefully and no conclusion regarding cause and effect can be made.

In conclusion, BV and PV is significantly increased in OW/OB compared to NW and this affects interpretation of iron biomarkers and provides insights into comparative iron metabolism in these groups. Our new proposed algorithm provides a good estimate of BV in OW/OB and could be used in further studies that require estimates of BV changes in OW/OB.
